# Circumferential Meniscal Reconstruction Using the Semitendinosus Tendon for a Medial Meniscal Posterior Root Tear

**DOI:** 10.1016/j.eats.2025.103495

**Published:** 2025-03-15

**Authors:** Takuya Ohno, Kei Nagasaki, Hiroki Ishikawa, Hiroki Okamura, Shogo Fujita, Mizuki Toura, Yoshifumi Kudo

**Affiliations:** a Department of Orthopedic Surgery, Nippon Koukan Hospital, Kanagawa, Japan; b Department of Orthopedic Surgery, Showa University School of Medicine, Tokyo, Japan

## Abstract

This article describes a surgical technique that addresses the limitations of existing approaches in managing meniscal extrusion after medial meniscal posterior root tear repair. Although traditional methods such as pullout repair and suture anchor repair are highly effective, they often struggle to adequately prevent meniscal extrusion, leading to suboptimal meniscal function restoration. Our method, which uses the semitendinosus tendon for circumferential joint capsule reinforcement, significantly reduces meniscal extrusion and enhances knee stability in patients with medial meniscal posterior root tears. Preliminary results suggest that this technique is superior to conventional methods in terms of preventing meniscal extrusion and restoring knee function.

A medial meniscal posterior root tear (MMPRT) contributes significantly to the development of subchondral insufficiency fractures of the knee and osteoarthritis (OA).^1^ This underscores the importance of restoring function effectively in this region of the meniscus. The meniscus is vital for equivalent load distribution within the knee, enhancing the contact area between the femur and tibia, which in turn reduces pressure.^2^ Without the meniscus, the contact area diminishes by 50% while the peak contact pressure increases by 200%.^3^

The MMPRT is characterized by a vertical radial tear or avulsion fracture within 10 mm of the medial meniscus’s footprint attachment.^4^ This type of tear raises the peak pressure. Medial complete root tears impair the conversion of axial load into hoop stress, leading to meniscal extrusion.^5^ Biomechanically, a complete root tear is equivalent to a total meniscectomy.^5^ Meniscal extrusion reduces the effective contact area across the articular surfaces and increases stress on the articular cartilage. It is also a predictor of cartilage loss in the tibiofemoral joint.^6^^,^^7^ Studies have indicated a significantly higher rate of cartilage volume loss in the medial tibiofemoral compartment in patients with meniscal extrusion compared with those without meniscal extrusion.^8^ Thus, functional repair of the MMPRT is essential.

Various techniques including transtibial pullout repair and suture anchor repair are available for MMPRT repair.^9^, ^10^, ^11^ However, these methods often fall short in maintaining hoop tension and preventing extrusion, resulting in inadequate meniscal function restoration.^12^ Ishikawa et al.^13^ recently proposed meniscal reconstruction for MMPRTs,^14^ and Li et al.^15^ found that the 2-year outcomes of medial meniscal posterior root reconstruction (MMPR-R) were superior to those of pullout repair. Additionally, Okamura et al.^16^ showed that although MMPR-R combined with opening-wedge high tibial osteotomy (OWHTO) had favorable outcomes, the improvement in meniscal extrusion was still insufficient.

Koga et al.^17^ implemented a centralization technique to address extrusion, whereas Kita et al.^18^ reported that favorable outcomes are expected with augmentation for radial tears. In this article, we present a method combining MMPR-R with the use of the semitendinosus tendon and an augmentation technique to restore physiological movement and effectively prevent meniscal extrusion.

## Surgical Technique

### Verification and Preparation of Tear

**Fig 1:**
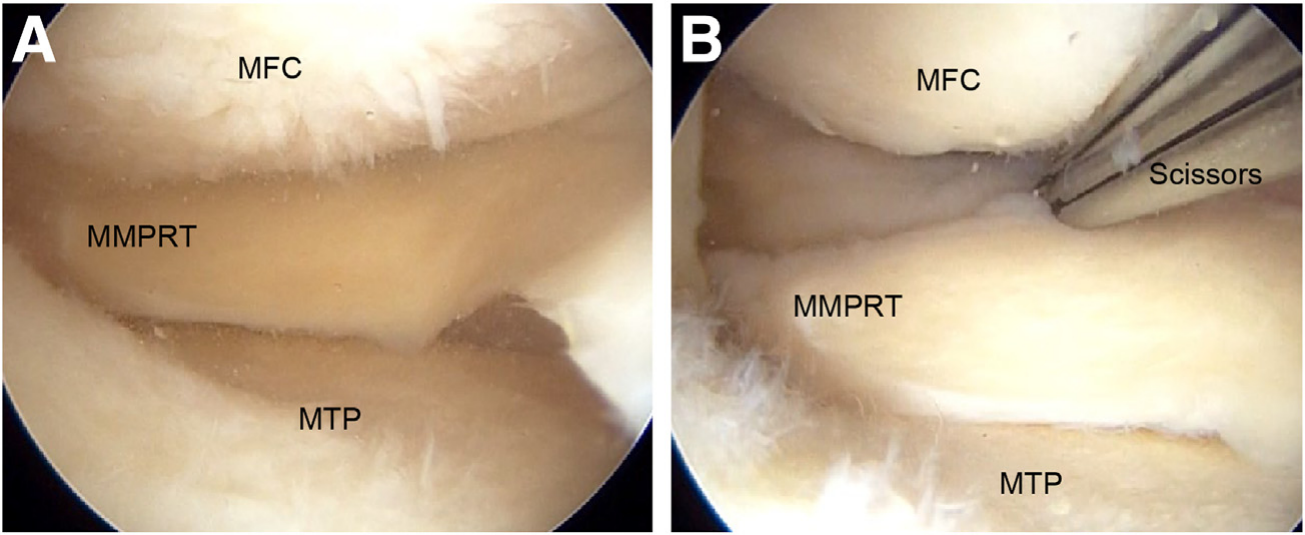
Medial meniscal posterior root tear (MMPRT) in right knee with patient in supine position. (A) An arthroscope is inserted through the anterolateral portal, and the MMPRT is identified and verified. (B) A surgical instrument such as a pair of scissors is used to create a hole in the posterior joint capsule approximately 1.5 cm from the attachment region of the medial meniscal posterior root. An Ethibond is passed through and brought outside the joint capsule. (MFC, medial femoral condyle; MTP, medial tibial plateau.)

The presence of an MMPRT is confirmed by probing through the anteromedial portal (Fig 1, Video 1). After verification, the torn section is debrided, and the tibial attachment, which is known for its rich blood supply, is prepared to facilitate meniscal reconstruction.

### Harvesting and Preparation of Autologous Semitendinosus Tendon

**Fig 2:**
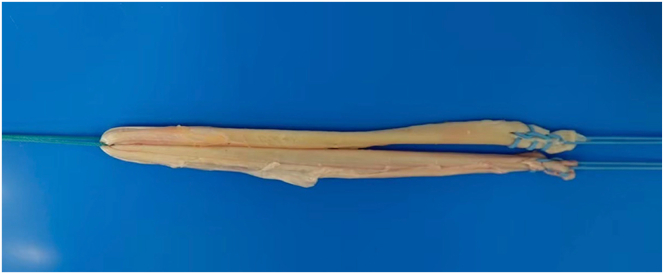
The 26-cm semitendinosus tendon is harvested and folded in half. The folded tendon is secured with Ethibond. To construct an extensive graft width, baseball-stitch sutures using Ti-Cron, coated with braided polyester, are placed at each end of the tendon’s double fold and connected side to side with Vicryl suture.

A 6-cm incision is made on the tibia’s medial aspect to access the semitendinosus tendon and simultaneously perform OWHTO. A 3-cm incision aligned with the sartorius muscle fibers that allows for tendon harvesting is made, after which the semitendinosus tendon is harvested. The superficial layer of the medial collateral ligament is detached using the pie-crusting technique with an 18-gauge injection needle at the medial epicondyle of the femur to create space between the femur and the meniscus (Table 1). These steps ensure adequate space for procedural maneuvers in the medial femorotibial joint. The harvested semitendinosus tendon, which is 26 cm long, is folded in half with Ethibond (Ethicon, Somerville, NJ). Baseball-stitch sutures are applied at both ends of the tendon’s double fold with Ti-Cron (Covidien, Dublin, Ireland) and secured with Vicryl suture (Ethicon) to construct a wide graft (Fig 2).

**Table 1:** Tips and Pitfalls

Tips
Adequate workspace in the medial tibiofemoral joint is ensured by releasing the superficial layer of the medial collateral ligament using the pie-crusting technique.
With the use of an anterior cruciate ligament reconstruction tibial tunnel guide, the tibial tunnel is precisely created at the anatomic footprint, and only the articular surface side is enlarged with a retrograde drill (AI Drill) to a depth of approximately 2 cm for graft end placement.
Regarding graft fixation, the graft ends must be pulled to apply the appropriate tension for joint capsule reinforcement.
The assist suture with the graft and the ruptured posterior root of the meniscus is performed after securing the graft itself.
Pitfalls
A 6-mm hole is carefully created in the posteromedial joint capsule to establish an entry point for the graft, ensuring that the popliteal artery and vein, as well as the tibial nerve, are not damaged during the process.
When passing the Ethibond outside the joint capsule and wrapping it circumferentially during graft passage and tensioned fixation, the surgeon must take care to avoid damaging the saphenous nerve and vein.

### Preparation for Joint Capsule Reinforcement

A 6-mm hole is created in the posteromedial joint capsule, approximately 1.5 cm from the attachment region of the medial meniscal posterior root (MMPR), using scissors, and the Ethibond is guided through the hole with a grasper via the anteromedial portal. The Ethibond is maneuvered externally along the capsule with a Pean clamp from the skin incision site, minimizing tissue damage. After the semitendinosus tendon graft is attached to the Ethibond, the graft is carefully pulled back through the capsule hole from outside to inside the joint.

### Preparation for Tibial Tunnel Creation

**Fig 3:**
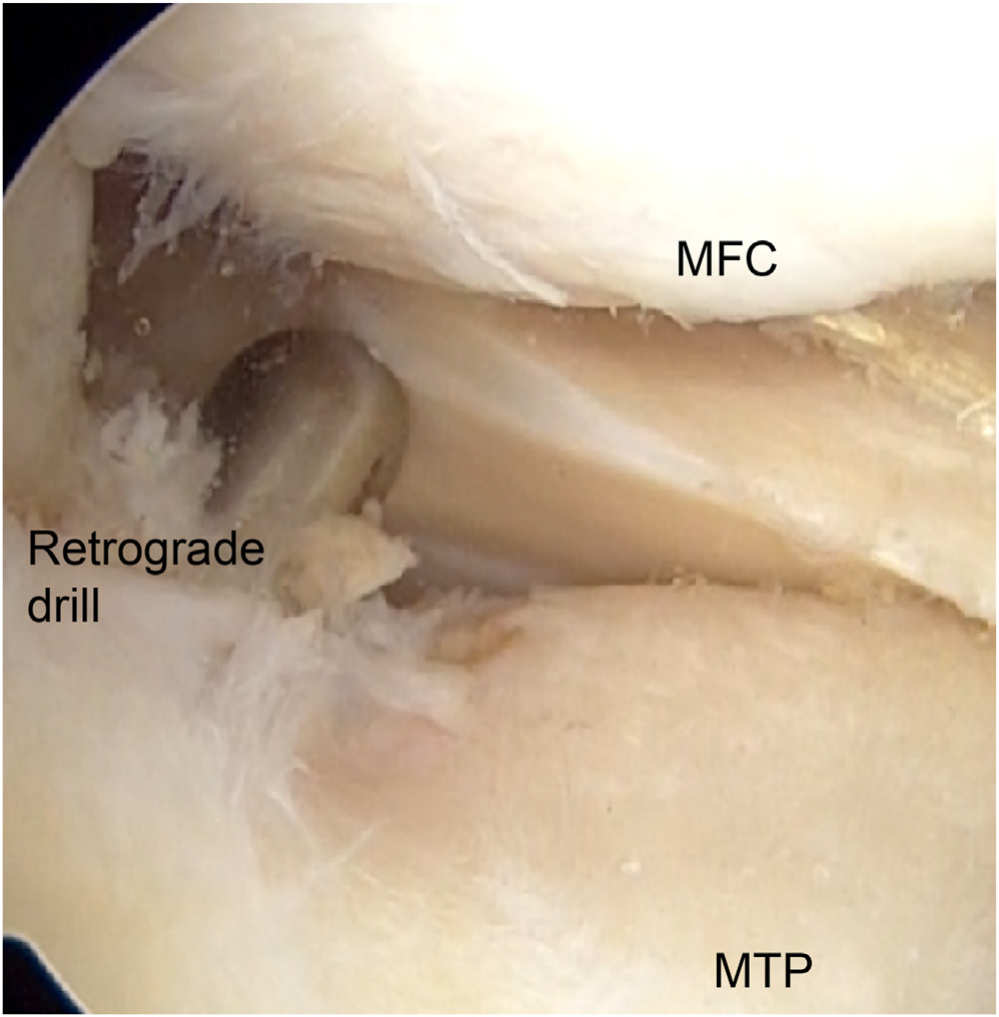
Right knee in supine position from anterolateral portal view. The tibial bone tunnel is formed with a 6.0-mm retrograde drill (AI Drill). The entrance of the bone tunnel should be addressed on medial meniscal posterior root attachment. (MFC, medial femoral condyle; MTP, medial tibial plateau.)

A 7-mm vertical incision is made 2 cm distal to the Gerdy tubercle on the tibia’s lateral aspect. Observation through the anterolateral portal allows for the confirmation of the tibial attachment of the MMPR and the identification of landmarks such as the medial intercondylar ridge and the anterior border of the posterior cruciate ligament. A guide pin is inserted through the lateral tibial incision using an anterior cruciate ligament reconstruction tibial tunnel guide (3M, St. Paul, MN), aimed at the MMPR’s tibial attachment (Table 1). After confirmation of the pin’s position, a tibial tunnel is created using a 4.5-mm drill, and the 2-cm depth of the joint side of the tunnel is enlarged to 6 mm with a retrograde drill (AI Drill; AI-Medic, Tokyo, Japan) to create space for the graft to fit securely (Fig 3, Video 1). In the present case, OWHTO is performed with a 62.5% mechanical axis percentage for a varus knee.

### Procedure for Graft Passage

**Fig 4:**
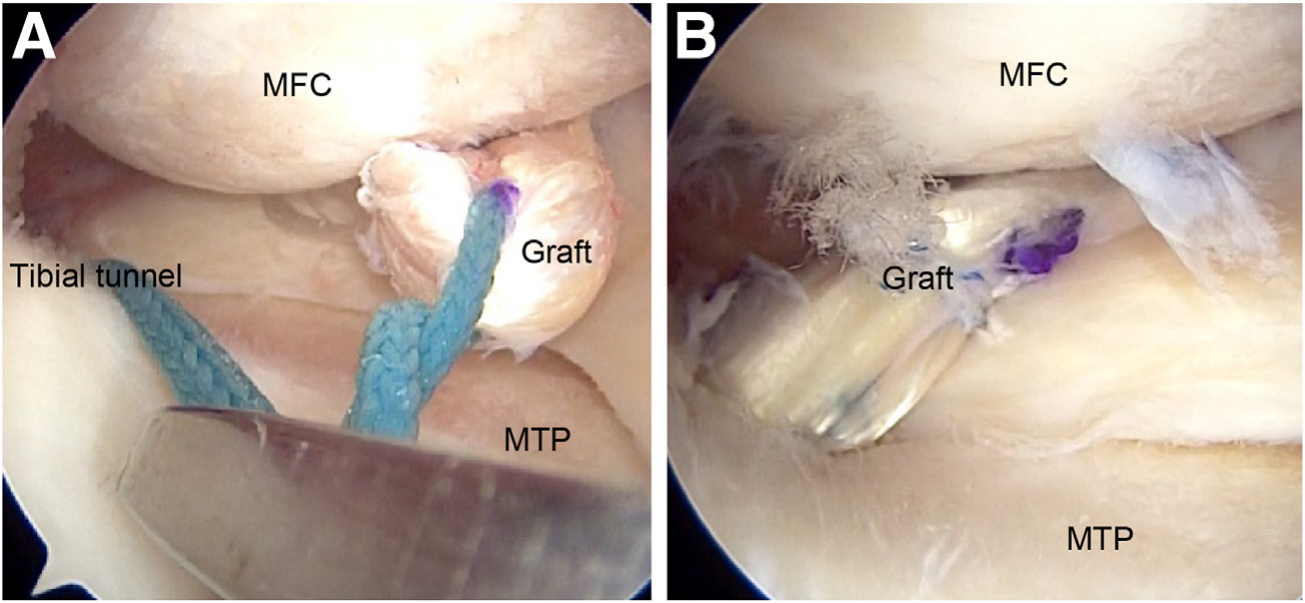
Right knee in supine position from anterolateral portal view. (A) The graft’s Ethibond is drawn out through the anteromedial portal and tied with the Ethibond from the tibial tunnel. By pulling the Ethibond exiting from the tibial tunnel, the graft is relayed into the tunnel using the shuttle-relay method. (B) Fixation is performed with an assist suture using Ultrabraid to facilitate adhesion with the graft and meniscus. (MFC, medial femoral condyle; MTP, medial tibial plateau.)

**Fig 5:**
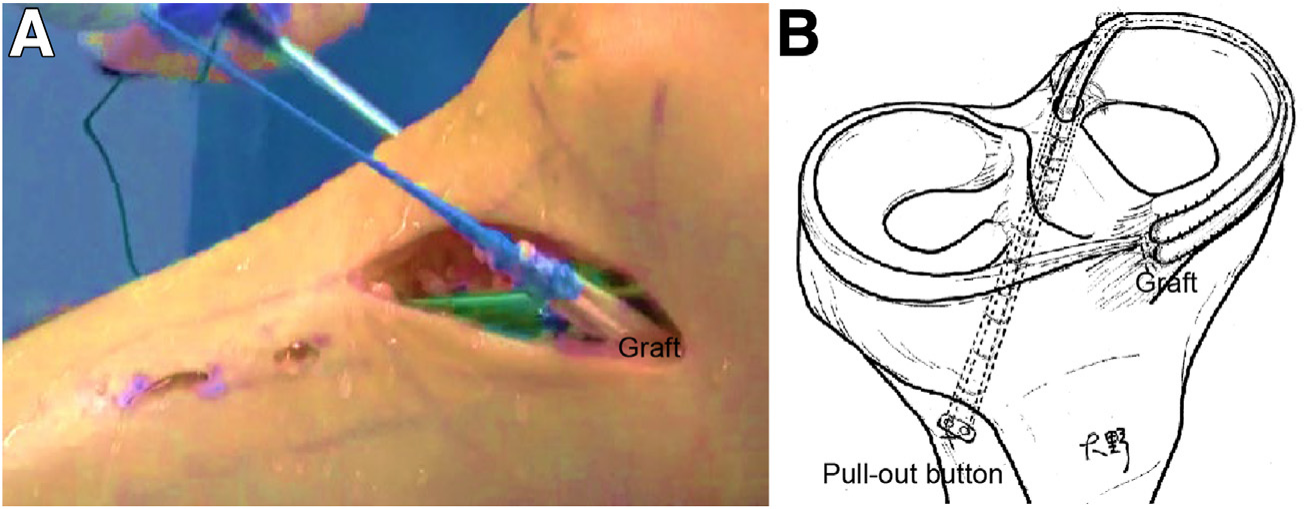
Right knee in supine position. (A) Medial outside view showing process of pulling Ethibond departing from tibial bone tunnel while making 1-cm incision in tibialis anterior muscle, securing pullout button (AI-Medic), routing graft circumferentially along joint capsule, and applying moderate tension. (B) Visual representation of reconstruction technique.

An Ethibond attached to a passing pin is inserted through the tibial tunnel and positioned as a loop. The Ethibond is maneuvered within the joint using a grasper introduced through the anteromedial portal and pulled out through the same portal. The graft’s Ethibond is also drawn out through the anteromedial portal. The graft’s Ethibond is securely tied to the Ethibond from the tibial tunnel, facilitating graft manipulation into the desired knee joint position. The Ethibond exiting the tibial tunnel is pulled to relay the graft into the tunnel using the shuttle-relay method (Fig 4, Video 1). The graft’s folded end is positioned within the bone tunnel, while the sutured ends wrap around the joint capsule and emerge through a medial skin incision on the tibia (Fig 5, Video 1).

### Fixation With Assist Suture

The graft’s folded-end Ethibond is secured using an artificial ligament fixture (pullout button; AI-Medic). Additionally, a polydioxanone suture (No. 2-0 PDS II; Ethicon) is passed through the reconstructed graft and posterior horn of the meniscus using the Meniscal Viper (Arthrex, Naples, FL), and Ultrabraid (Smith & Nephew, Watford, England) is replaced via the shuttle-relay method to reinforce fixation on the femoral and tibial sides of the graft and meniscus. Ti-Cron attached to the graft end is pulled to apply the appropriate tension for joint capsule reinforcement (Table 1). The graft end is then secured with Vicryl suture to construct a wide graft and is sutured to the anterior coronary ligament of the knee.

### Postoperative Assessment and Rehabilitation Protocol

**Fig 6:**
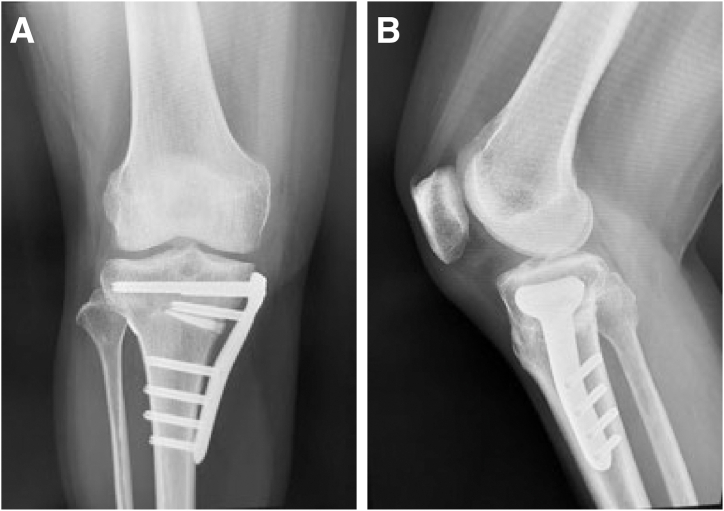
Postoperative radiographic images of right knee. (A) Frontal view. (B) Lateral view. The pullout button (AI-Medic) is secured on the lateral side of the tibia.

**Fig 7:**
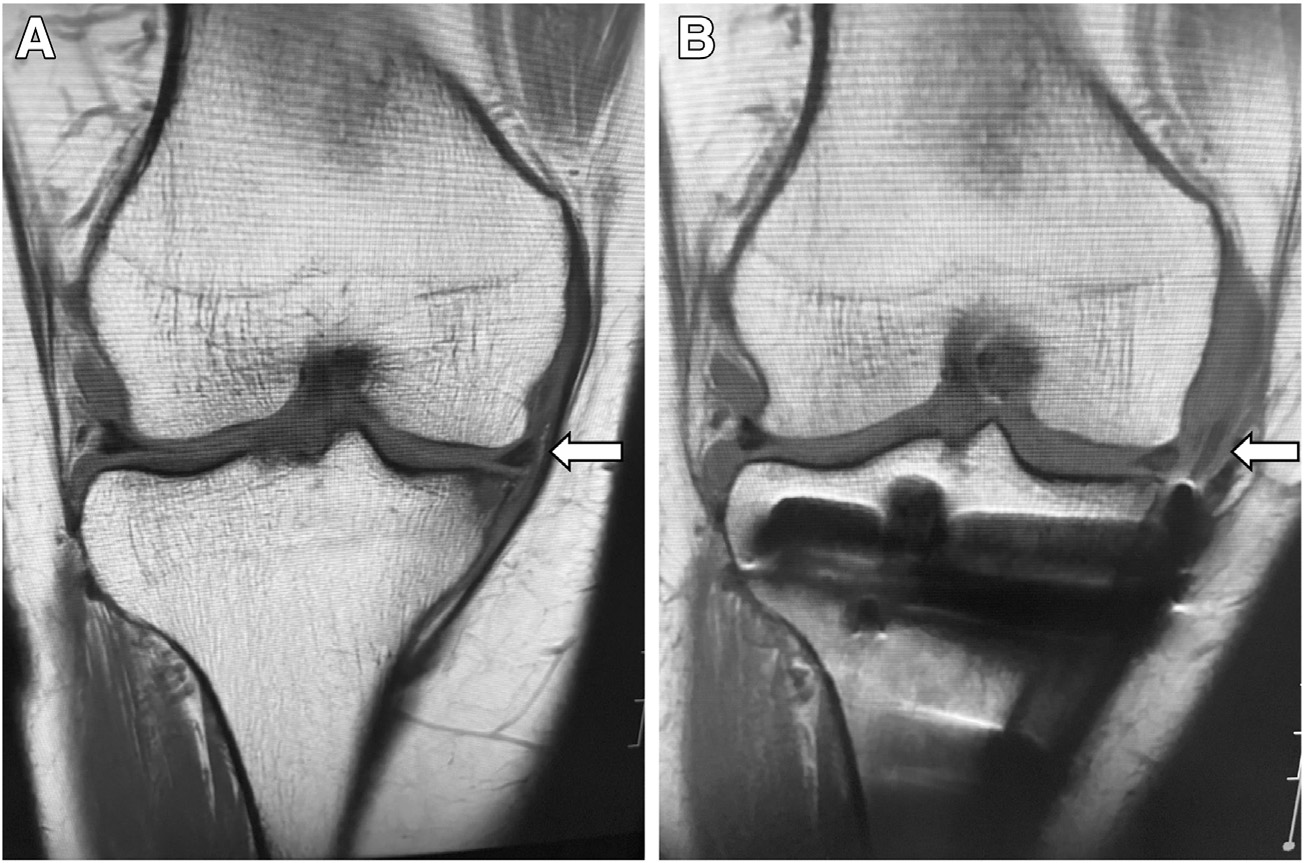
Preoperative (A) and postoperative (B) magnetic resonance images of right knee. Postoperatively, as indicated by the white arrows, the extrusion of the medial meniscus is corrected, and the graft created on the lateral side of the joint capsule is visible.

After surgery, radiography (Fig 6) and magnetic resonance imaging (Fig 7) are performed and a structured rehabilitation protocol is implemented. Patients initially adhere to a non–weight-bearing regimen for the first 2 weeks, transitioning to partial weight bearing by the third week, and increasing gradually to full weight bearing by the sixth week. Knee flexion is limited to 90° for the first 4 weeks, gradually increasing to 130° by the twelfth week. Full knee flexion and squatting are permitted 3 months postoperatively, with clearance for sports activities 8 months after surgery.

## Discussion

Restoring hoop tension in MMPRTs is vital for slowing joint degeneration and OA progression.^1^ Although various fixation techniques exist, achieving moderate meniscal function recovery through repair alone is challenging.^19^ Our technique aims to prevent meniscal extrusion and restore the physiological function of the knee.

Meniscal extrusion disrupts normal load distribution in the knee, increasing stress on the articular cartilage and potentially accelerating OA.^2^^,^^8^ Surgical reduction of extrusion is difficult, even with advanced techniques, and some degree of extrusion often persists.^20^^,^^21^

Studies have shown the aggravation of meniscal extrusion after fixation in many cases. Chung et al.^11^ found that 59% of patients had increased extrusion 1 year after transtibial pullout repair. Moon et al.^22^ also observed an increase in extrusion postoperatively. These findings highlight the limitations of traditional techniques and the need for improved procedures. Koga et al.^17^ examined an arthroscopic centralization technique for medial meniscal extrusion, using knotless anchors to effectively reduce extrusion and restore function. Kita et al.^18^ described a technique that enhances meniscal healing by augmenting circumferential fibers rather than merely stabilizing the damaged area. Okamura et al.^16^ used the gracilis tendon for MMPR-R, showing potential in restoring hoop function but not fully addressing extrusion. Building on these ideas, we developed a technique to wrap the semitendinosus tendon graft around the joint capsule, effectively preventing extrusion and allowing for meniscal repositioning. In our case, magnetic resonance imaging and radiography revealed satisfactory results, and the patient’s knee pain was resolved (Fig 7).

The choice of graft is crucial. A study by Rönnblad et al.^23^ on autologous semitendinosus tendon grafts for meniscal transplantation showed favorable early results. Tucker et al.^24^ found the semitendinosus tendon superior in load distribution and tensile strength compared with synthetic alternatives, supporting its use in meniscal reconstruction. Furthermore, it is well known in anterior cruciate ligament reconstruction that both the semitendinosus tendon and gracilis tendon exhibit excellent bone-tendon adhesion.^25^

Further biomechanical considerations include the double-folded semitendinosus tendon, which provides additional strength and stability and potentially reduces rerupture risk.^26^ Combining OWHTO with reconstruction reduces the femorotibial angle and posterior tibial slope, alleviating stress on the MMPR and preventing graft damage.^27^ The tendon, doubled and secured into a hole on the tibial side of the medial meniscus’s posterior root attachment, is routed along the joint capsule to reinforce it. The circumferential reinforcement of the joint capsule may also distribute load more evenly, reducing cartilage degeneration.^2^^,^^5^ Moreover, the lateral placement of the tibial bone tunnel and pullout button prevents the graft from making a killer turn during placement, which consequently minimizes graft damage and enhances graft longevity.^28^^,^^29^

In conclusion, circumferential meniscal reconstruction using the semitendinosus tendon for MMPRTs is a relatively simple technique that has shown favorable results in preventing extrusion and restoring knee function (Table 2). In the future, long-term studies and biomechanical assessments with defined patient selection criteria and optimized rehabilitation protocols will be essential to confirm the effectiveness of this method as a standard treatment for MMPRTs (Table 2).

**Table 2:** Advantages and Disadvantages of Technique

Advantages
Functional meniscal repair requires improved hoop function and prevents extrusion. Reconstruction using the semitendinosus tendon with circumferential reinforcement of the joint capsule achieves this.
Reconstruction with autologous tendons avoids immune responses and raises no ethical concerns. Additionally, using the semitendinosus tendon promotes favorable bone-tendon healing.
When this procedure is performed simultaneously with high tibial osteotomy, the tendons can be harvested through the same skin incision used for osteotomy.
Disadvantages
The concept of this surgical procedure is straightforward; however, a certain level of skill and technique is required to perform it effectively.
Complications may arise at the donor site.

## Disclosures

All authors (T.O., K.N., H.I., H.O., S.F., M.T., Y.K.) declare that they have no known competing financial interests or personal relationships that could have appeared to influence the work reported in this paper.

## Supplementary Data

Video 1 Comprehensive, step-by-step demonstration of arthroscopic technique for reconstructing medial meniscal posterior root tear using combined approach of posterior root reconstruction and circumferential joint capsule reinforcement. The procedure is performed on the right knee with the patient in the supine position. Visualization is achieved through the anterolateral portal, while the working portals include the anteromedial portal. The video covers the entire process, beginning with identification and assessment of the medial meniscal root tear. It then moves on to preparation of joint capsule reinforcement and creation of the tibial tunnel, followed by passage and fixation of the graft. The final segment of the video shows fixation of the reconstructed graft onto the anterior coronary ligament using an assist suture, highlighting the structural integrity achieved through the procedure.
